# Tyrosine 416 Is Phosphorylated in the Closed, Repressed Conformation of c-Src

**DOI:** 10.1371/journal.pone.0071035

**Published:** 2013-07-26

**Authors:** Sevgi Irtegun, Rebecca J. Wood, Angelique R. Ormsby, Terrence D. Mulhern, Danny M. Hatters

**Affiliations:** 1 Department of Biochemistry and Molecular Biology and Bio21 Molecular Science and Biotechnology Institute, The University of Melbourne, Melbourne, Australia; UCL Institute of Neurology, United Kingdom

## Abstract

c-Src kinase activity is regulated by phosphorylation of Y527 and Y416. Y527 phosphorylation stabilizes a closed conformation, which suppresses kinase activity towards substrates, whereas phosphorylation at Y416 promotes an elevated kinase activity by stabilizing the activation loop in a manner permissive for substrate binding. Here we investigated the correlation of Y416 phosphorylation with c-Src activity when c-Src was locked into the open and closed conformations (by mutations Y527F and Q528E, P529E, G530I respectively). Consistent with prior findings, we found Y416 to be more greatly phosphorylated when c-Src was in an open, active conformation. However, we also observed an appreciable amount of Y416 was phosphorylated when c-Src was in a closed, repressed conformation under conditions by which c-Src was unable to phosphorylate substrate STAT3. The phosphorylation of Y416 in the closed conformation arose by autophosphorylation, since abolishing kinase activity by mutating the ATP binding site (K295M) prevented phosphorylation. Basal Y416 phosphorylation correlated positively with cellular levels of c-Src suggesting autophosphorylation depended on self-association. Using sedimentation velocity analysis on cell lysate with fluorescence detection optics, we confirmed that c-Src forms monomers and dimers, with the open conformation also forming a minor population of larger mass complexes. Collectively, our studies suggest a model by which dimerization of c-Src primes c-Src via Y416 phosphorylation to enable rapid potentiation of activity when Src adopts an open conformation. Once in the open conformation, c-Src can amplify the response by recruiting and phosphorylating substrates such as STAT3 and increasing the extent of autophosphorylation.

## Introduction

c-Src signaling controls many cellular events such as cell growth, proliferation, differentiation, motility and cell adhesion [1]. The kinase activity of c-Src depends on whether the protein is in the more expanded “open” active conformation or in the more compact “closed” repressed conformation [2]. Phosphorylation of Y527 facilitates the formation of the closed conformation by enabling high affinity binding of the SH2 domain to the C-tail. This interaction, as well as binding between the SH3 domain and the SH2-kinase linker, creates a compact structure that represses kinase activity. Dephosphorylation of Y527 releases SH2 binding to the C-tail leading to a more open conformation with far greater kinase activity [3], [4]. Open active c-Src can be induced by the mutation Y527F which impairs binding of SH2 and hence impedes formation of the closed repressed state [5]. Conversely, mutating the C-tail at residues Q528E, P529E, G530I, to mimic a high affinity c-Src SH2 ligand induces a constitutively closed state, as reported previously for the Src family member Hck [6].

Y416 resides in the activation loop of the kinase domain and its autophosphorylation is commonly invoked in models of c-Src regulation as a key step leading to high c-Src activity [7], [8], [9], [10]. This is supported by several lines of evidence. One is that v-Src from the Rous sarcoma virus, which is a constitutively active c-Src homologue, is phosphorylated at Y416 to a greater extent than c-Src [11]. Another is that the mutation Y416F reduces kinase activity [10], [12], [13]. A third is that c-Src displays a capacity to autophosphorylate Y416 and that in the phosphorylated state has a higher kinase activity [14], [15], [16]. Crystal structures of c-Src and related kinase Lck are consistent with phospho-Y416 stabilizing the conformation of the activation loop in a manner permissive for substrate binding [3], [17].

Because Y416 phosphorylation correlates with greater activity, phosphorylation levels of Y416 have been used as a determinant of c-Src catalytic activity [18], [19], [20]. During our studies, we found that Y416 phosphorylation occurred to a different extent to phosphorylation of a substrate, STAT3 [21], [22]. As a consequence we investigated the basis of this effect in more detail. Here we describe our findings, and in particular the finding that c-Src is appreciably phosphorylated at Y416 when in the closed repressed conformation and at the same time unable to phosphorylate STAT3.

## Materials and Methods

### DNA constructs

cDNA of the protein sequences for human c-Src (NP_005408) with the C-terminal extension GSGSDPPVAT were synthesized using human-optimized codons (Mr Gene, Life Technologies). The sequences were cloned into the pT-REx vector (Life Technologies) using standard cloning procedures. The monomeric Emerald fluorescent protein (EGFP with mutations S72A, N149K, M153T, I167T, A206K [23], [24]) was fused directly to the C-terminus of the linker using standard PCR-mediated cloning procedures. The open (Y527F) and closed (Q528E, P529E, G530I) mutations were introduced using QuickChange mutagenesis (Agilent Technologies). The kinase-dead (K295 M) mutants of c-Src were generated by QuickChange Lightning Site Directed Mutagenesis (Agilent Technologies). The truncated c-Src(Δ1–63) variants were generated by PCR mediated cloning strategies and subcloned back into the pT-REx sequence retaining the same fusion sequence to Emerald. The GFP with the Y66L mutation to render the protein non-fluorescent was generated in the pT-REx vector as previously described [25]. The mKate2-F sequence [26] was commercially synthesized (GeneArt) with human optimized codons and appended at the C-terminus with the sequence SGLRTKLNPPDESGPGCMSCKCVLS, which includes C-terminal 20 amino acid farnesylation signal sequence from the c-HA-Ras protein [27], [28]. The mKate2-F sequence was subcloned into pT-Rex vector (Invitrogen) using standard PCR-mediated cloning procedures.

### Cell culture and transfections

All experiments were performed in AD293 cells, which were maintained in Dulbecco's modified Eagle medium (DMEM) supplemented with 10% FCS, 2 mM glutamine, 200 U/mL penicillin and 200 µg/mL streptomycin. The culture flasks were kept at 37°C in a humidified incubator with 5% atmospheric CO_2_. For imaging by confocal microscopy, 1.1×10^5^ cells were transfected in solution with 0.96 µg vector DNA and 1.5 µL Lipofectamine 2000 reagent in serum reduced Opti-MEM (Life Technologies) and plated into tissue culture treated optical-grade 8-well chamber slides that were pre-coated with 0.01% (w/v) poly-L-lysine for 30 min, washed with water and dried for 2 h. The cells were incubated for 24 h expression. For Western blotting, 1×10^6^ cells were plated into 25 cm^2^ tissue culture flasks. After 24 h cells were rinsed in serum reduced Opti-MEM and then transfected using 2 µg of DNA and 5 µL of Lipofectamine 2000. After 6 h transfection, the media was replaced with supplemented DMEM. For analytical ultracentrifugation, 4×10^6^ cells in 75 cm^2^ tissue culture flasks were transfected with 24 μg DNA and 60 μL Lipofectamine 2000 as described for Western blotting, with the media replaced after 7 hours. The cells were incubated for 24 h expression.

### Western Blotting

Cells were harvested in 5 mL DMEM with a cell scraper. The cell suspension was transferred into the 15 mL plastic tube and centrifuged at 3315×*g* for 3 min at room temperature. The pellet was resuspended in 5 mL ice-cold phosphate-buffered saline (PBS) by gently pipetting up and down. The cells were again pelleted by centrifugation at 3315×*g* for 3 min at 4 °C. The pelleted cells were lysed with 300 µL of RIPA buffer (150 mM NaCl, 50 mM Tris, 1 mM EDTA, 1% (v/v) Triton X-100, 1% (w/v) sodium deoxycholate, 0.1% (w/v) SDS, 0.2% (w/v) sodium fluoride, 0.2% (w/v) sodium orthovanadate, 20 U/mL benzonase and Complete EDTA-free protease inhibitor (Roche Diagnostics)) on ice for 40 min. Aliquots were then snap-frozen in liquid nitrogen and stored at −80°C. Total cellular protein concentration was determined in triplicate using a BCA protein assay using bovine serum albumin as the standard, according to manufacturer's instructions (Thermo Scientific). Protein samples were resolved by 12% (v/v) polyacrylamide SDS-PAGE and transferred to PVDF membrane at 100 V for 1 h in 25 mM Tris, 192 mM glycine, 20% (v/v) methanol, pH 8.3, using a Criterion Blotter Transfer System (Bio Rad). The membrane was blocked with 5% (w/v) skim milk powder or 3% (w/v) BSA for the phospho-specific antibodies in PBS-T (PBS, 0.1% (v/v) Tween-20) for 1 h at room temperature or overnight at 4°C. Primary antibodies were added to the blocking buffer for 2 h at room temperature or overnight at 4°C. Antibodies were used at 1∶10,000 dilution for GFP (#A-6455, Invitrogen), 1∶1,000 dilution for STAT3 (#610189, BD Biosciences), 1∶1,000 for STAT3 pY705 (#9131, Cell Signaling), 1∶500 c-Src (#N-16, Santa Cruz Biotechnology), 1∶7,500 for c-Src pY416#1 (#PK1109, Calbiochem), 1∶7,500 for c-Src pY416#2 (#2101, Cell Signaling), 1∶10,000 for α-tubulin (Invitrogen). The membrane was then washed four times over 30 min with PBS-T before probing with horseradish peroxidase-conjugated secondary antibodies for 1 h at room temperature. The membrane was washed four times over 30 min with PBS-T. The protein bands were visualized using enhanced chemiluminence according to manufacturer's instruction (Sigma-Aldrich) and digital acquisition by a LAS-3000 imager (Fujifilm).

### Analytical ultracentrifugation

Transfected cells were harvested by scraping off the plate in 5 mL DMEM and placed into a tube. Cells were pelleted (500×*g*; 5 min room temperature) and pellet was resuspended on ice in 1 mL ice cold native lysis buffer (NLB) (20 mM Tris pH 8, 2 mM MgCl_2_, 1% (w/v) Triton X-100, Complete-EDTA-free protease inhibitor (Roche Diagnostics), 0.2% (w/v) sodium fluoride, 0.2% (w/v) sodium orthovanadate, 20 U/mL benzonase, 1 mM PMSF). Cells were mechanically lysed on ice by extrusion through an 27 Gauge needle 20 times. 5 M NaCl was added to a final concentration of 150 mM. Lysate was maintained on ice until analytical ultracentrifugation. Total protein concentration was measured using a BCA assay and adjusted to 0.5 mg/mL with NLB. Lysate was loaded into Charcoal-Epon 12 mm thick Velocity quartz-window centerpieces, prechilled at 4°C overnight, and centerpieces placed into a prechilled AnTi-8 rotor also prechilled at 4°C overnight. Samples were analyzed with an XL-A analytical ultracentrifuge (Beckman Coulter) equipped with a fluorescence detection module (AVIV) and 488 nm laser at 10°C. Continuous radial fluorescence scans were collected at a rotor speed of 3,000 rpm for 2 h. The rotor speed was increased to 40,000 rpm and a further 200 scans were collected. The sedimenting boundaries were fitted to a model describing the sedimentation of a distribution of sedimentation coefficients c(s), which were converted to mass units, c(M), using the program SEDFIT [29]. Data were fitted using a regularization parameter of F = 0.95, TI noise correction and partial specific volume of 0.73 g/mL. The frictional ratios and meniscus position were floated. Buffer viscosity was estimated as 0.013336 Poise, and density 1.00665 g/mL based on buffer composition using SEDNTERP (John Philo, Thousand Oaks, CA).

## Results

**Figure 1:**
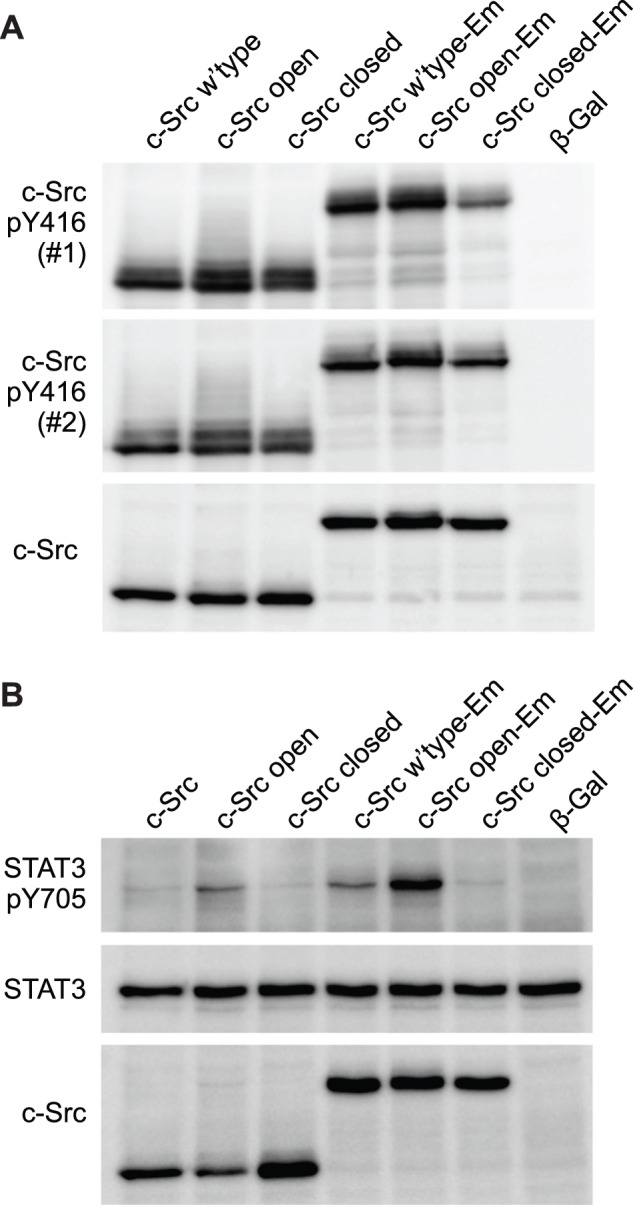
Y416 is phosphorylated in closed, repressed c-Src. AD293 cells were transfected with the indicated constructs and after 24h analysed by Western Blots as shown. Key: Em, Emerald fluorescent protein; closed, Q528E, P529E, G530I mutant of c-Src; open, Y527F mutant of c-Src; β-gal, β -galactosidase in same vector as c-Src constructs **A.** The blot was standardized to c-Src levels. Phospho-Y416 was detected with two different antibodies: #1, cat. PK1109 from Calbiochem; #2, cat. 2101 from Cell Signaling. **B.** The blot was standardized to endogenous STAT3. Blots were performed twice and showed consistent results.

First we probed the basal levels of Y416 phosphorylation of c-Src transfected into AD293 cells by Western Blot. As part of our experiments, we included c-Src fused to the GFP derivative Emerald. Fusion of c-Src to GFP with a few spacer residues has been used to study c-Src cell biology without disrupting key functional properties of c-Src [30]. Wild-type c-Src and mutants that constitutively adopt an open, active conformation (Y527F) or a closed, repressed conformation (Q528E, P529E, G530I) were investigated alone and in fusion to Emerald (Fig 1). After first standardizing the amount of lysate to c-Src levels, blots were analyzed with two different antibodies for c-Src phospho-Y416. Both antibodies bound specifically to all forms of c-Src as evidenced by no immunoreactivity to a negative control of cells transfected with β-galactosidase (Fig 1A). The extent of reactivity of the antibodies was qualitatively greater for the open conformation than the closed, and the wild-type was intermediate. The pattern of phospho-Y416 staining was similar in the context of the Emerald tag and without the tag, suggesting that the Emerald does not perturb the fundamental regulatory mechanisms of c-Src. A notable feature of the data was that the repressed, closed conformation of c-Src had a substantial capacity to be phosphorylated at Y416.

To further investigate the underlying kinase activity of c-Src under our conditions and the relationship to Y416 phosphorylation, we examined the phosphorylation status of Y705 on STAT3, which is a downstream target of c-Src [21], [22]. For this experiment, protein loads on Western Blots were standardized to endogenous STAT3 levels using STAT3 immunoreactivity (Fig 1B). Blots were reprobed with an antibody for phospho-Y705 on STAT3, which revealed a greater level of reactivity for the open conformation of c-Src, consistent with open c-Src mutant displaying greater kinase activity. The closed conformations of c-Src conferred only trace levels of STAT3 Y705 phosphorylation, relative to the open conformation, and the wild-type was intermediate for both the Emerald-tagged and untagged c-Src proteins. These findings suggest that c-Src Y416 and STAT3 Y705 phosphorylation loosely correlate with each other, but that there is also a high basal Y416 phosphorylation when STAT3 phosphorylation is largely absent.

**Figure 2:**
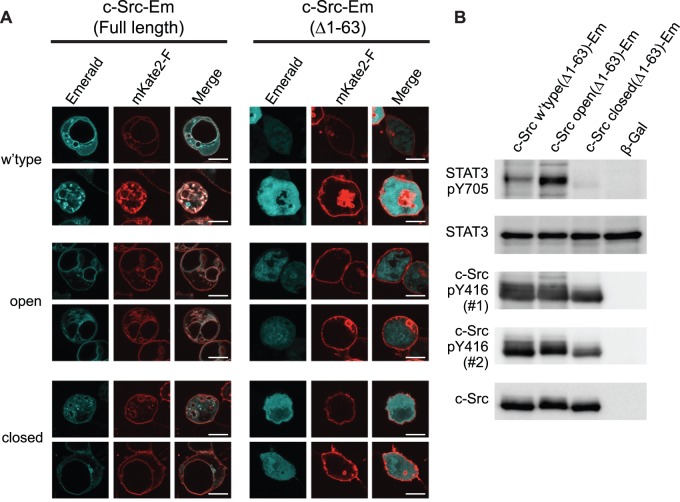
Phosphorylation of Y416 in the closed, repressed conformation is driven by core kinase domains. **A.** Confocal micrographs of c-Src expressed as fusions to Emerald in AD293 cells in either full length or truncated form lacking the first 63 residues (Δ1–63), which contains the unique domain and myristoylation sequences. Cells were co-transfected with mKate2-F, which is a fluorescent protein containing a farnesylation signal to localize it to membranes. Turquoise shows c-Src-Em, red shows mKate2-F. Scale bar, 10 µm. Images are representative of three independent experiments. **B.** Western Blots of AD293 cells transfected with the indicated constructs for 24 h with 10 µg total protein lysate. Phospho-Y416 was detected with two different antibodies: #1, cat. PK1109 from Calbiochem; #2, cat. 2101 from Cell Signaling. Key: Em, Emerald fluorescent protein; closed, Q528E, P529E, G530I mutant of c-Src; open, Y527F mutant of c-Src; β-gal, β -galactosidase in same vector as c-Src constructs. Experiments were performed at least twice, which showed consistent results.

We next investigated whether the Y416 phosphorylation patterns were governed by the minimal unit of c-Src for regulating activity: the SH2, SH3, kinase, and C-tail domains [3], [31]. For this experiment, we deleted the intrinsically disordered N-terminal region (residues 1–63), which includes the unique region and a myristoylation sequence that selectively targets c-Src to the membrane, receptors and binding partners [32], [33], [34], [35]. c-Src(Δ1-63)-Emerald localized primarily in the cytosol, whereas the full length counterpart localized more extensively to the edge of the cell and internal membranous structures, consistent with its previously reported membrane/endosomal localization patterns ([36]; Fig 2A). Despite the altered cellular localization, c-Src(Δ1–63) in wild-type, open and closed forms conferred similar patterns of STAT3 Y705 phosphorylation to that of the full length protein (Fig 2B). Y416 phosphorylation was also still abundant in the closed form of c-Src(Δ1–63) (Fig 2B). Collectively, these results indicated the core functional unit of the SH2, SH3 and kinase domains of c-Src is sufficient to phosphorylate targets, such as STAT3, and itself by autophosphorylation of Y416. Furthermore, Y416 autophosphorylation persisted when these core domains were locked in the closed conformation.

**Figure 3:**
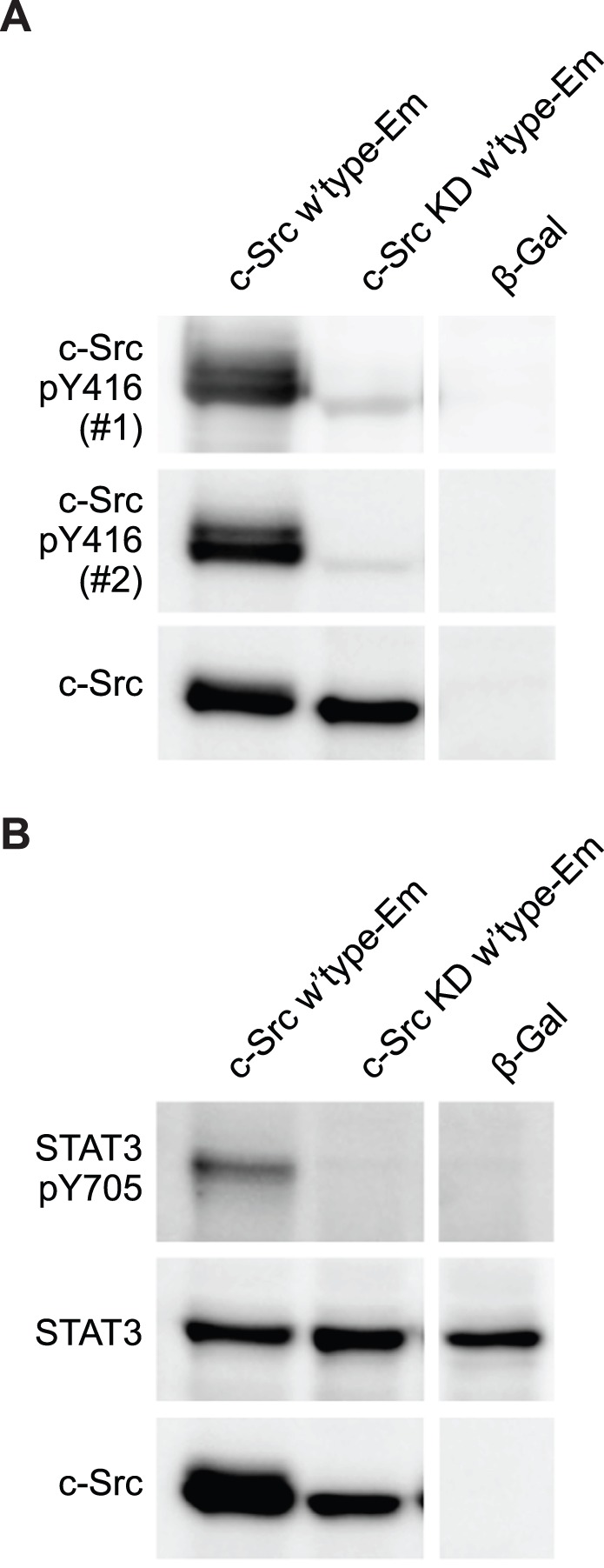
Phosphorylation of Y416 requires kinase activity and is sustained upon suppression of activity. AD293 cells were transfected with the indicated constructs and analysed by Western Blot 24 h post-transfection. Blots were performed at least twice, which showed consistent results; a single representative is shown. Key: KD, kinase dead K293 M mutant; Em, Emerald fluorescent protein; closed, Q528E, P529E, G530I mutant of c-Src; open, Y527F mutant of c-Src; β-gal, β -galactosidase in same vector as c-Src constructs. **A.** Western Blot standardized for c-Src levels. Phospho-Y416 was detected with two different antibodies: #1, cat. PK1109 from Calbiochem; #2, cat. 2101 from Cell Signaling. **B.** Western Blot standardized for STAT3 levels.

To confirm whether the Y416 phosphorylation observed in the closed conformation was dependent on autophosphorylation or occurred from endogenous kinases, we investigated the basal Y461 levels of phosphorylation in a catalytically inactive mutant of c-Src (K295 M) [37], [38]. The K295 M mutant abolished Y416 (Fig 3A) and STAT3 Y705 phosphorylation (Fig 3B), suggesting that endogenous proteins do not lead to the phosphorylation of Y416 and that Y416 phosphorylation arises directly through autophosphorylation.

**Figure 4:**
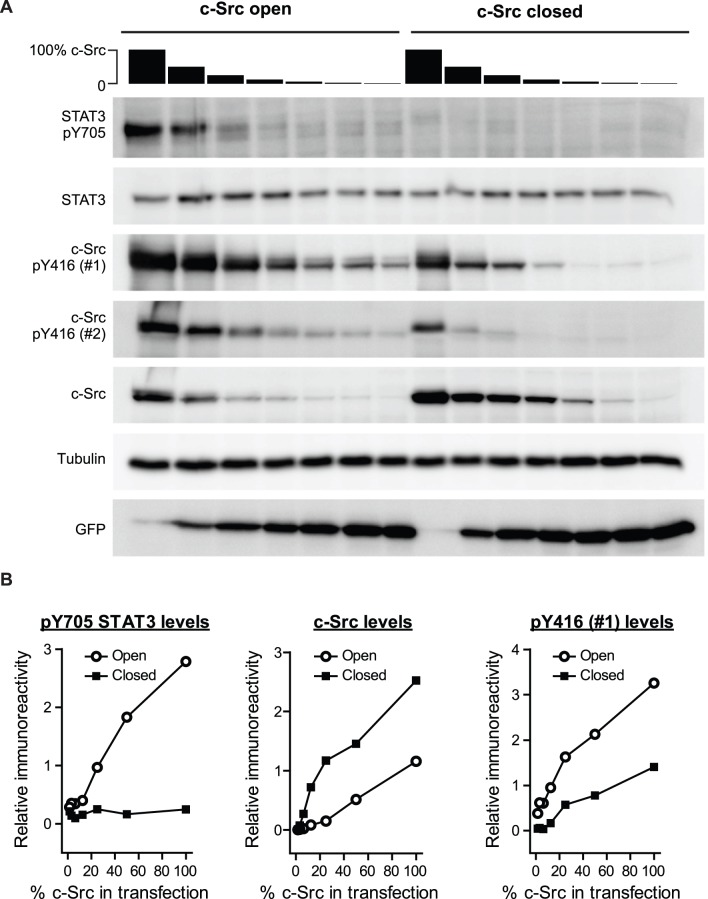
Expression-level dependence of c-Src on Y416 phosphorylation. **A.** Western Blots of AD293 cells transfected with c-Src variants for 24 h standardized to β-tubulin. C-Src was transfected at different doses by adjusting the proportion of DNA used in transfections from 100% to 1.56% supplemented with a vector expressing non-fluorescent Y66L GFP derivative. Key: closed, Q528E, P529E, G530I mutant of c-Src; open, Y527F mutant of c-Src. Phospho-Y416 was detected with two different antibodies: #1, cat. PK1109 from Calbiochem; #2, cat. 2101 from Cell Signaling. **B.** Densitometry analysis of the Western Blot data in panel A for STAT3 phospho-Y705 immunoreactivity, c-Src immunoreactivity and c-Src phospho-Y416 immunoreactivity with antibody #1 (cat. PK1109). Blots shown are representative of three independent experiments. Densitometry analysis is for the blots shown.

We next examined the dependency of Y416 phosphorylation on expression level of c-Src. Various doses of constitutively open and closed c-Src variants were transfected into AD293 cells with DNA titrated against a plasmid expressing a non-fluorescent Y66L GFP derivative [25] to maintain a constant total load of DNA in the transfection. Western blots showed a dose dependent increase in STAT3 Y705 phosphorylation for constitutively open c-Src, whereas there was no phosphorylation for closed c-Src (Fig 4A). Despite the lack of STAT3 phosphorylation for closed c-Src, there was a pronounced level of baseline Y416 phosphorylation – especially at high levels of expression which suggests autophosphorylation can be decoupled from substrate phosphorylation (Fig 4A and 4B). Open c-Src displayed a proportionally far higher level of STAT3 and autophosphorylation than closed c-Src. It is also noteworthy that the extent of Y416 phosphorylation for the open state seemed far more extensive at lower c-Src expression levels than the highest levels (Fig 4B). One explanation for this result is that Y416 phosphorylation in the open state is potentiated by c-Src interactions with endogenous ligands, which would become saturated at very low c-Src levels. This mechanism is consistent with open c-Src becoming fully activated by Y416 phosphorylation when in complex with substrates.

**Figure 5:**
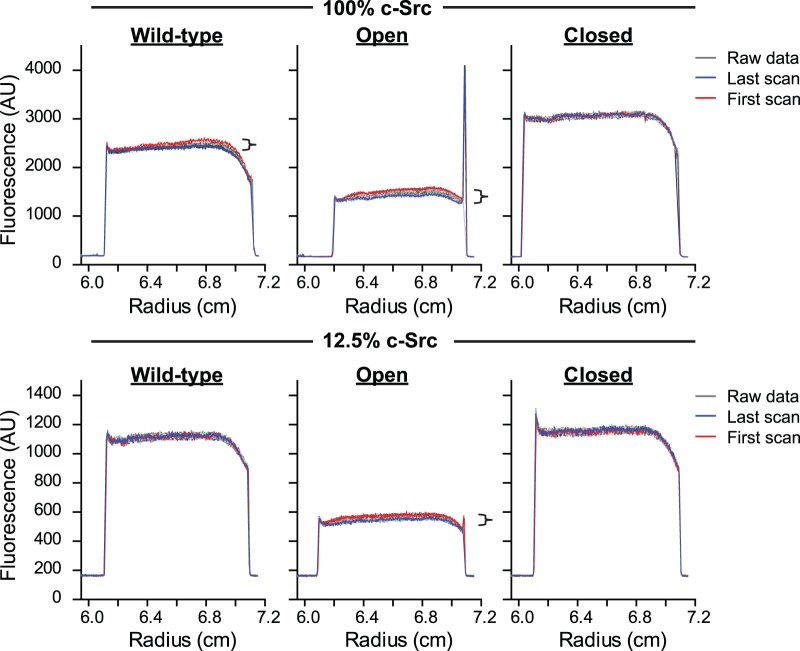
Open c-Src forms a minor proportion of high mass complex in cell lysate. Sedimentation velocity data at low speed (3,000 rpm) by analytical ultracentrifugation of cell lysates of AD293 cells transfected with c-Src-Emerald is shown as raw data (gray incrementing lines), with the first scan shown in red and the last scan in blue. Data are representative of three independent experiments. Cells were transfected with two different doses of c-Src: either 100% c-Src in transfection, or 12.5% c-Src with the remainder of the DNA supplemented with a non-fluorescent Y66L derivative of Emerald. Under these conditions low mass forms of c-Src-Emerald such as monomers and dimers will not sediment. Brackets indicate the loss of fluorescence in the supernatant due to pelleting of high mass complex (>∼1000 S).

**Figure 6:**
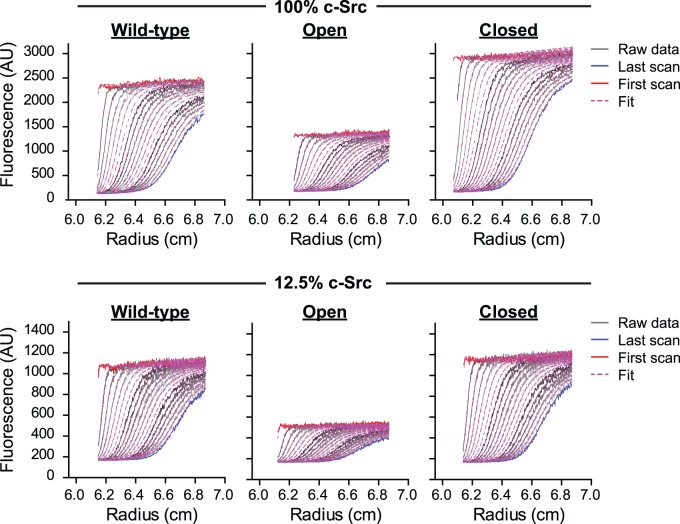
Open and closed c-Src predominately form low mass oligomers in cell lysate. Sedimentation velocity data at high speed (40,000 rpm) by analytical ultracentrifugation of cell lysates of AD293 cells transfected with c-Src-Emerald is shown as raw data (gray incrementing lines), with the first scan shown in red and the last scan in blue. Data shown are representative of three independent experiments. Fits to a c(s) model are shown in magenta dashed lines. Cells were transfected with two different doses of c-Src: either 100% c-Src in transfection, or 12.5% c-Src with the remainder of the DNA supplemented with a non-fluorescent Y66L derivative of Emerald.

**Figure 7:**
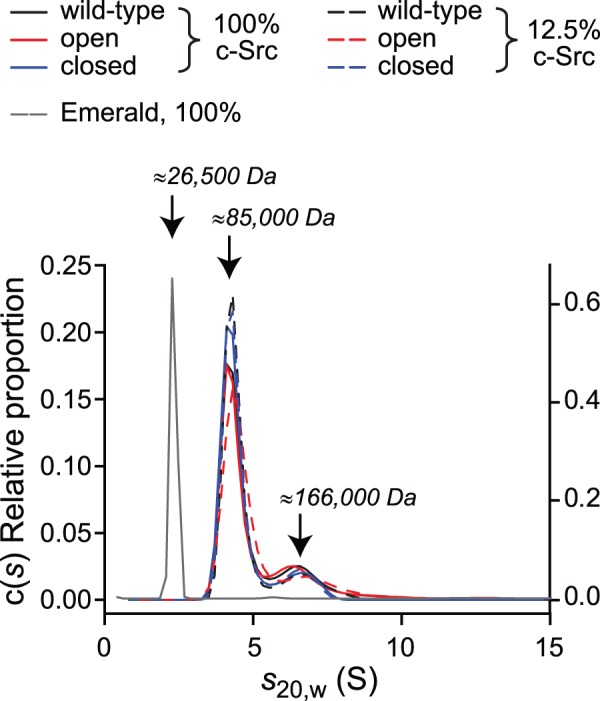
Open and closed c-Src form predominately monomers and dimers in cell lysate. Size distributions of the c(s) fit to the data in Figure 6, as well as a control of Emerald-transfected cell lysate, are shown. Corresponding masses based on c(M) size distributions are shown with the arrow for the two major species. Three independent experiments produced consistent results; data shown are for a single experiment.

**Figure 8:**
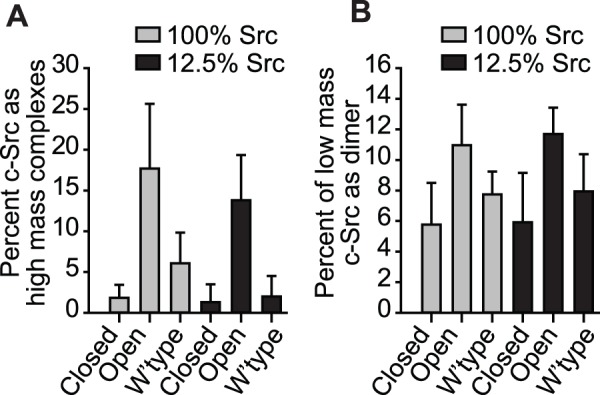
Open c-Src forms high mass complexes and dimers to a greater extent than the closed and wild type forms. **A.** Percent of c-Src that forms high mass complexes (>∼1000 S). Values were calculated as percent of fluorescence intensity lost from the supernatant at 6.8 cm radius between the first and last sedimentation velocity scans at low speed (3000 rpm). Loss of intensity is indicated by brackets in Figure 5. (Data shows mean ± S.D., n = 3). There was a significant difference between open and closed (P = <0.001), wild-type versus open (P = 0.001) but not wild-type versus closed (P = 0.362) as assessed by two-way ANOVA and Holm-Sidak pairwise comparison. There was no difference between 100% and 12.5% transfection conditions (P = 0.206; two-way ANOVA). **B.** Percent of low mass c-Src that exists in dimers. Values are calculated as the percent of material greater than 5.2 S from size distribution of c(s) fits to high speed (40,000 rpm) sedimentation velocity data. (Data shows mean ± S.D., n = 3). There was a significant difference between open and closed (P = 0.007) but not wild-type versus closed (P = 0.183) or wild-type versus open (P = 0.059) as assessed by two-way ANOVA and Holm-Sidak pairwise comparison. There was no difference between 100% and 12.5% transfection conditions (P = 0.760; two-way ANOVA).

One possibility to explain why Y416 phosphorylation occurs in the closed repressed state in a manner decoupled from substrate phosphorylation is that autophosphorylation is driven at least partly by self-association. To investigate the state of association of c-Src, we examined the sedimentation coefficient of c-Src-Emerald fusions directly in cell lysate using an analytical ultracentrifuge equipped with a fluorescence detection module, which we previously used to quantitate the oligomeric state of Emerald-tagged proteins in cell lysate [25]. Lysate was prepared at two different doses of c-Src in transfections: 100% c-Src DNA versus 12.5% c-Src DNA with 87.5% Y66L GFP to maintain a constant DNA load. We first assessed the lysate at low centrifugal speed (3,000 rpm) for evidence of high mass complexes for transfections (Fig 5). Most material did not sediment under these conditions as indicated by an unchanging boundary plateau over time. The open forms of Src and to a lesser extent the wild-type revealed a minor (∼10%) decrease in the fluorescence intensity of the plateau over the timecourse of the experiment (several h) (Fig 5). This was not observed with the closed mutant. One explanation for this result is that the open form of c-Src, but not the closed, can recruit other molecules into high mass macromolecular complexes that are pelleted at this low centrifugal force. When the rotor speed was increased (40,000 rpm), all samples formed a family of sedimenting boundaries over time, indicating the samples comprised predominately of low mass forms of c-Src-Emerald (Fig 6). Fits to a c(s) size distribution model, which describes the sedimentation of a distribution of non-interacting molecules revealed two predominant masses (*s*_20,w_ = 4.3 S and 6.5 S) (Fig 6 and 7). The corresponding c(M) masses for these sedimentation coefficients are 85 kDa and 166 kDa, which are consistent with monomer and dimer (*c.f.* 87,766 Da for monomer mass based on amino acid composition) (Fig 7). These data thus support a model whereby c-Src dimerization mediates Y416 phosphorylation even when c-Src is in a closed state. It is noteworthy that the open state more extensively formed dimers and greater masses than the wild-type and closed states based upon the fitted c(s) data converted to proportions of c-Src molecules in each mass (Fig 8). This result is consistent with small number of the open conformations binding to other ligands or in dynamic exchange with larger masses that would not be clearly resolved by c(s) analysis. This raises the possibility that the potentiation of c-Src activity and autophosphorylation is mediated through larger clustering patterns or interactions with other ligands.

## Discussion

Here we show that as per previous studies, levels of Y416 phosphorylation is correlated to the open active conformation of c-Src [7], [8], [9], [10], [14], [15], [16]. The greatest extent of Y416 phosphorylation occurred at lower levels of c-Src expression, which is consistent with Y416 phosphorylation occurring cooperatively when c-Src is engaged with ligands or other cellular complexes that are concentration limiting with increasing levels of c-Src. In addition, we observed an appreciable level of basal Y416 phosphorylation in the closed repressed conformation of c-Src. Under these conditions, c-Src did not lead to STAT3 phosphorylation, suggesting that kinase activity for autophosphorylation can be decoupled from substrate phosphorylation. Our findings are consistent with previous reports that a constitutively closed YEEI mutant of the Src-family member Hck could undergo autophosphorylation [39], [40].

Prior studies have suggested that Src family kinases have a more complex multi-state conformational regulation than a two-state “open” and “closed”. Specifically, it was reported that the constitutively closed YEEI Hck mutant could be activated by the high-affinity SH3 ligand Nef [40], which suggests an intermediate activation state between fully closed and fully open. In addition, further conformational states are implicated by studies showing that displacement of the SH2 domain of Hck in the high affinity linker (HAL) mutant can lead to activation without SH3 displacement [41]. Therefore, it remains plausible that the basal c-Src autophosphorylation arises due an intermediate active conformation of c-Src that still has the C-tail locked to the SH2 domain. Because STAT3 is recruited in an SH3-dependent manner to c-Src [42], an intermediate SH3-displaced state could result in STAT3 phosphorylation by c-Src that still has the C-tail locked to the SH2. However, our results demonstrate that these conformations are not sufficiently populated to enable efficient STAT3 phosphorylation.

We propose that c-Src autophosphorylation may occur partly by the dimerization of c-Src, which we showed occurs for both open and closed c-Src, but which trends to higher amounts for the open state and at higher levels of expression. Hence, both open and closed c-Src participate in monomer-dimer equilibria that seems to influence Y416 autophosphorylation.

The autophosphorylation of Y416 in the closed state in a concentration-dependent manner has intriguing implications for the mechanisms of c-Src regulation. These findings point to a model whereby Y416 autophosphorylation could act as a mechanism to prime locally concentrated closed c-Src for high activity upon kinase opening by secondary events. This mechanism of control of c-Src activity is conceivably important for rapidly modulating and amplifying c-Src activity at focal adhesions, where c-Src is locally enriched and engaged with focal adhesion kinase, which binds to c-Src through the SH2 domain in the open conformation [43], [44]. This process is also likely to not require membrane-anchored self-association, since we observed substantial autophosphorylation of c-Src in the closed state lacking the membrane anchoring myristoylation sequence. However the lipid anchor would be important for localized differences in activity where different ligands would also be co-enriched. Also, recent data for Src family member Lck suggests clustering on the cell surface is greater for the open conformation than the closed upon T cell receptor signaling [45], which indicates that lipid anchoring plays important roles in coordinating the clustering processes.

The second implication is that because STAT3 phosphorylation is much more dependent on c-Src being in the open conformation than autophosphorylation, then priming of Y416 will not be sufficient to activate substrate phosphorylation. Previous studies have suggested that STAT3 phosphorylation requires c- Src to have a functional SH3 domain [42]. Therefore, open c-Src, where the SH3 will be more accessible, should interact strongly with STAT3, leading to its phosphorylation; while closed c-Src, where the SH3 is more effectively locked away in the intermolecular interaction with the SH2-kinase linker, should fail to recognize STAT3, leading to little, if any, phosphorylation. Thus a second layer of control over c-Src kinase activity is possible whereby closed, repressed c-Src can be recruited into local clusters, which primes c-Src through Y416 phosphorylation. Secondary recruitment of substrate can hence occur without phosphorylation until c-Src is switched into the open conformation by dephosphorylation of Y527. Since the open conformation also sustains Y416 phosphorylation, this second step would positively reinforce c-Src activity.
